# Effects of diets containing grape pomace on the growth, nutrition indices, and the quality traits of common carp (*Cyprinus carpio*)

**DOI:** 10.1002/fsn3.3614

**Published:** 2023-08-10

**Authors:** Barzan Mahmoodi, Ali Aberoumand, Saeed Ziaei‐nejad, Sadra Seyyedi

**Affiliations:** ^1^ Department of Fisheries Behbahan Khatam Alanbia University of Technology Behbahan Iran; ^2^ Behbahan Khatam Alanbia University of Technology Behbahan Iran

**Keywords:** carp, fillet, fish, grape pomace, nutrients

## Abstract

The fish diet is one of the essential factors in the development of aquaculture. The purpose of present study was to evaluate using grape pomace as a main feed ingredient on growth performance, body chemical composition, survival rate, and morphological indices of the carp (*Cyprinus carpio*). 200 fish with an average weight of 7 ± 0.4 g were randomly distributed in four tanks for total of 56 days. The fish were fed with a diet containing 5%, 10%, and 15% grape pomace in different feeding groups designated as G1, G2, and G3. The average daily weight gain (g), weights gain (g), and specific growth rate (%) were significantly higher (*p* < .05) in G3 as compared to G1 and G2 groups. The lowest feed conversion ratio was recorded in G3 group. The morphological indices, condition factors, viscerosomatic index, and hepatosomatic index were significantly higher in G3 group as compared to other treatments. The protein, fat, moisture, and ash contents in the *C. carpio* fillets were significantly influenced by feeding rate. The results showed that grape pomace had a positive effect on growth, survival, and nutritional indices in the carp fish. According to the obtained results, grape pomace (150 g/kg food) can be considered as the diet component for the carp fish.

## Graphical abstract

The average daily weight gain (g), weight gain (g), and specific growth rate (%) were significantly higher (*p* < .05) in T3 as compared to T1 and T2 groups. The lowest feed conversion ratio was recorded in T3 group. The morphological indices, condition factors, viscerosomatic index, and hepatosomatic index were significantly higher in T3 group as compared to other treatments. The protein, fat, moisture, and ash contents of the whole fillets of the *Cyprinus carpio* were significantly influenced by feeding rate. The results showed that grape pomace had a positive effect on growth, survival, and nutritional indices in the carp fish. According to the obtained results, grape pomace (150 g/kg food) can be considered as the diet component for the carp fish. The percentage of body weight gain of common carp under the influence of different amounts of grape pomace (control treatment), T1 (5% treatment), T2 (10% treatment), T3 (15% treatment). Results shown that grape pomace 15% concentration found highest of effect on the fish growth.

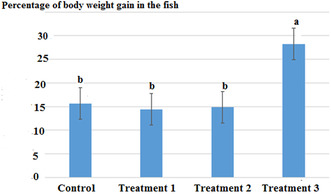

## 1 INTRODUCTION

Aquaculture is considered to be the fastest‐growing food production industry with an average growth rate of more than 7.7% per year in the past decades; the majority of aquaculture production comes from Asia. Around 600 aquatic species are bred in captivity in about 190 countries for production in fish farming systems with different input intensities and technological sophistications (Almabrok et al., 2018).

Aquaculture plays an important role in raising marine species for food security and nutrition. The increase in the food production industry leads to the reduction of overexploitation of wild stocks and the prevention of ecosystem degradation. Fish nutrition management is essential to minimize costs and maximize growth in sustainable aquaculture practice (Ahmad et al., 2018; Daet, 2019; Sangeeta et al., 2018). The use of useful and economical foods is important for the demand for fish feed and to optimize the ingredients for efficient use (Pena et al., 2019).

Medicinal plants and grape pomace have been used in aquaculture nutrition due to the presence of various active compounds such as flavonoids, alkaloids, phenolics, pigments, steroids, terpenoids, enhancing appetite and combating microbes, and other stressful factors (Almabrok et al., 2018).

Common carp (*Cyprinus carpio*) is one of the most important fish species in aquaculture and one of the economically important fish species that is cultivated mainly in Asia and Europe. The global production of cultured *C. carpio* accounts for about 6.14% of global aquaculture production. It is also cultivated commercially in other parts of the world due to its fast growth rate, easy cultivation, and high food efficiency ratio, and it consumes less fish meal and fish oil than other species (Mohamed et al., 2018).

According to FAO (2018), among food‐producing sectors, aquaculture industry is the fastest‐rising sector in the world. The occurrence of fish disease and use of chemicals such as antibiotics are the most determining factors in aquaculture. However, it is a hard reality that use of chemicals and antibiotics in aquaculture may result in antibiotic resistance in bacteria and finally leads to environmental hazards. Therefore, discovering environment‐friendly products and developing their appropriate application plans are of utmost importance currently. In this direction, studies are being undertaken to find out suitable organic products that are environment‐friendly, sustainable, and cost‐effective. Several medicinal plants have the potential positive effects on growth performance, survival rate, and immune system activation in fish (Bilen et al., 2020).

One of the problems that arise in aquaculture is high production costs, which is mostly due to feed costs. Feed is a limiting factor, especially in fish farming, because so far, fishmeal in feed has not been successfully replaced by another ingredient. Since fishmeal also comes from wild fish populations, the lack of fish stocks also affects the availability of feed and thus has a large impact on the sustainability of aquaculture. Studies in recent decades have focused on natural products to overcome these problems. Among these natural products, phytochemicals are gaining attention due to their many health benefits. Phytochemicals are bioactive compounds of plant origin that show antiviral, anticancer, growth‐stimulating, antibiotic, antioxidant, immune system stimulating, and anti‐inflammatory effects. Phytochemicals have been successfully shown to promote growth in various fish species. The use of phytochemicals as feed additives is currently a popular field in aquaculture and has been investigated in recent years. In addition, more comprehensive research is needed to evaluate the industrial application of phytochemicals on a larger scale (Taştan & Salem, 2021).

Inadequate nutritional factors are known to cause various diseases in fish. Adequate nutrients in the diet directly affect fish health and disease prevention. Therefore, nutritional management strategies play an important role in the success of aquaculture. Since the last decade, the use of medicinal plants and their bioactive compounds has been widely used to prevent diseases and maintain fish health in aquaculture. The bioactive compounds of these plants have various beneficial effects for animals (Mohamed et al., 2022).

When the carp fed with plant material will have a high growth rate, this growth rate is less when fed with animal food (Dawood et al., 2020). Grape is rich in phenols which are distributed in its pomace. The grape pomace is a source of bioactive compounds, polyphenols, indigestible fiber, fatty acids, and minerals due to its high protein value as fertilizer (Arslan et al., 2018; Lange et al., 2014; Nowshehri et al., 2015; Zhai et al., 2014). The dietary grape pomace application in aquaculture is to improve the growth rate, antioxidant status, and disease resistance in various fish and shellfish species (Arslan et al., 2018; Lange et al., 2014). The purpose of this study was to investigate the effects of grape pomace on growth and survival indices, nutritional indices, and body composition of the carp fish.

## 2 MATERIALS AND METHODS

### 2.1 Preparation of fish

The present research was conducted from 2019 to 2020 in Laboratory of Fisheries at Khatam Alanbia University of Technology in Behbahan, Iran. In this project, 200 fish with an average weight of 7 ± 0.4 g and a length of 6.5 ± 0.5 cm were prepared from the Center of Preservation and Reconstruction of Native Fish Stocks of Shahid Maleki in Ahvaz, Iran. To transfer the fish from Ahvaz to the University Aquaculture Center, plastic bags were used in two layers, in which one‐third was filled with water and two‐thirds were filled with oxygen, and its lids were tightly closed. The fish kept in another laboratory for a week to prevent possible disease transferred to the main workshop. The fish were kept and disinfected, during fed commercial during the adaptation period.

### 2.2 Feeding/grow‐out period

This study was performed with four treatments (triplicate for each). After adaptation period, fish were transferred to 300 L tanks, which contained 250 L of water, and aerated using stone embedded in each tank. In this experiment, 15 fish were randomly placed in each tank, and then feeding period was started in four treatments (control, 5%, 10%, and 15% grape pomace in fish feed). Water was exchanged at the rate of 20% of water volume every day to maintain the best water quality. However, before distributing the fish in each tank, biometry indices were measured. The fish were analyzed for each tank in triplicate.

### 2.3 Water quality management

In order to prevent pollution and reduce water quality, every day the bottom of the tanks was cleaned and feces and food residues were removed from the tank, and also 20% of the water in the tank was changed.

### 2.4 Water quality analysis

In the present study, the physicochemical parameters of water were measured. Water samples were collected from different distinct locations. The parameters analyzed included pH and temperature, electrical conductivity (EC), and dissolved oxygen (DO), following the methods described by Bassi et al. (2014) and American Public Health Association (APHA) (2017). The samples were collected in acid‐soaked (5% HNO_3_) and rinsed polypropylene bottles and kept at 40°C prior to laboratory analysis. DO and pH were measured on‐site, while the remaining parameters were assessed in the laboratory. Physicochemical factors of water such as temperature and oxygen were checked daily, and pH, EC, and other factors were checked weekly. The water temperature of each tank during the period was on average 24°C, pH was 7.5, and DO was 7.5 mg per liter, and the water tanks were dechlorinated for 24 h before use. The light regime during the growth period was observed as 12 h of light and 12 h of darkness.

### 2.5 Preparation of diet

To feed the fish, a commercial feed especially for carp fish with Fara Daneh brand was used. In order to prepare a diet based on different concentrations of grape pomace, first, 1 kg of commercial feed prepared for each treatment was powdered with an electric grinder. The powdered feed was divided into three parts. The grapes with species (*Vitis vinifera*) were prepared from Behbahan market and in laboratory were dried in an oven at 60°C for 6 h, then were powdered by a grinder, and then were mixed with powdered commercial feed in concentrations of 5%, 10%, and 15% (w/w). Because according to the reports of the researchers, it was possible to the grow fish in this range of dried grape pomace concentrations. The control group was prepared from commercial feed without grape pomace powder. Then, an equal volume of distilled water was added to it and mixed well. Then, feed pellets were prepared using a digital grinder. After drying in the oven at a temperature of 60°C for 12 h, the feed pellets were prepared and crushed using a sieve with the appropriate size made for feeding the fish. To prevent oxidation, the prepared feed was kept at 4°C until their use.

### 2.6 Fish feeding

First, the total weight of the fish in each tank was measured and according to their average body weight, the amount of feed required for daily feeding (based on 3% of body weight) was determined and then, fish feeding was done three times a day. According to biometric results, which were performed every 15 days, the amount of feed required was calculated according to body weight gain of 3%.

### 2.7 Final sampling and bioassay

This experiment was performed based on a completely randomized design. The duration of the experiment was 56 days. Because 56 days was the suitable period for fish feeding and growth. Each treatment group had three replicates, and the number of fish in each group was 50. Feeding was stopped for 24 h to empty the gastrointestinal tract of fish and to evaluate the nutritional, growth, and survival indices. The total length of the fish was measured using a biometric board, and its weight was measured using a digital scale with an accuracy of 0.01 g. Also, the number of fish losses, if observed during the growing period, was recorded for each tank separately which used at the end of the period to calculate the percentage of fish survival. No pathogens were observed during the total 56 days in fish growing period.

### 2.8 The growth and survival indices

At the end of 56 days, feeding period to evaluate growth and survival indices such as average body weight gain, daily weight gain, body weight gain percentage, specific growth rate percentage, status index (Zokaeifar et al., 2012), and survival percentage (Geurden et al., 1996) and nutritional indices such as feed conversion ratio (FCR), liver index, and visceral index (VSI; Fuchs et al., 2015; Peixoto et al., 2016; Portz et al., 2001), the following formulas were used:

$$\text{Weight gain}\;\left(\mathrm{WG},g\right)=\text{Final body weight}‐\text{initial body weight}.$$

$$\text{Average daily weight gain},\left(\text{ADWG},g/\mathrm{day}\right)=\left\{\left(\text{Final body weight}\;\left(g\right)‐\text{Initial body weight}\right)/\text{time}\;\left(\text{days}\right)\right\}.$$

$$\text{Body weight gain}=\text{final weight}\;\left(g\right)‐\text{initial weight}\left(g\right)/\text{initial weight}\left(g\right)\times 100$$

$$\mathrm{SGR},\%/\mathrm{day}=\left(\ln\;\left(\text{final body weight}\right)‐\ln\;\left(\text{initial body weight}\right)\times 100\right)/\text{time}\;\left(\text{days}\right).$$

$$\text{Condition factor}\;\left(\mathrm{CF},g/{\mathrm{cm}}^{3}\right)=\left(\text{Body weight}\left(g\right)/{\text{Body length}}^{3}\left(\mathrm{cm}\right)\right)\times 100.$$

$$\text{Survival rate}\;\left(\mathrm{SR},\%\right)=\left(\mathrm{No}.\;\text{of fish survived}/\mathrm{No}.\;\text{of fish released}\right)\times 100.$$

$$\text{Feed conversion ratio}\;\left(\mathrm{FCR}\right)=\text{Feed given}\;\left(g\right)/\text{Weight gain}\;\left(g\right).$$

$$\text{Hepatosomatic index}\;\left(\mathrm{HSI}\%\right)=\text{liver weight}/\text{body weight}\times 100.$$

$$\text{Viscerosomatic index}\;\left(\mathrm{VSI}\%\right)=\text{weight of viscera and associated}\;\mathrm{fat}\;\text{tissue}/\text{body weight}\times 100.$$

### 2.9 Chemical composition analysis

The standard methods (AOAC, 2005) were used to analyze the proximate composition of the fish tissue samples. All experiments were performed in triplicate. The proximate compounds were protein, fat, moisture, and ash. To powder the samples, around 150 g of pre‐prepared fillet was placed in the oven at 70°C for 24 h to dry completely. The samples were then cooled and powdered by a grinder machine. They were then placed in separate containers and finally transferred to the laboratory for analysis.

### 2.10 Moisture measurement

Moisture content was determined by drying 20 g of powdered sample in the oven (*Model*: Memmert of Germany) at 105°C for 24 h to reach a constant weight (AOAC, 2005). After removing the dish from the oven, it was placed in desiccator for 30 min to be cooled down. The sample was then weighed, and moisture content was determined using the following formula:

$$\text{Moisture content}=\text{primary weight of the sample}\;\left(g\right)-\text{secondary sample of the sample}\;\left(g\right)\times 100/\text{primary weight of the sample}\;\left(g\right)$$

### 2.11 Determination of crude fat

The 5 g of the sample was weighed with a digital scale with an accuracy of 0.01 g. The sample was placed in filter paper and after weighing, it was placed in the extractor section of the Soxhlet apparatus. The 100 mL of petroleum ether was placed into the balloon; the apparatus was connected to refrigerant, and the extraction was carried out by a heater at 40–60°C for 8 h. The distillation of the solution continued until the balloon was free of solvent (Portz et al., 2001). Then, samples were dried in a hood. The crude fat content was calculated using the following equation:

$$\begin{array}{c} \text{Crudefat percentage}=\text{Initial sample weight}–\text{free}\;\mathrm{fat}\;\text{sample weight}/\\ \;\text{Initial sample weight}\times 100 \end{array}$$

### 2.12 Determination of crude protein

The sample's crude protein content was determined using the Kejaledal method. To determine the crude protein in the samples, 1 g of the sample was placed into the digestion balloon, and 150 mL of concentrated sulfuric acid with a catalyst was added to each balloon. After placing the balloons in the apparatus, the sample is boiled at low temperature for about 30 min to remove the foam, and then the temperature is increased to digest the sample. Sample digestion took time about 4 h. After digesting the samples and cooling, distilled water was added to each balloon and placed in the titration section of the Kjaldal device and titrated with 0.1 N normal sulfuric acid. Total nitrogen was determined by the Kjaldal method and then multiplied by 6.25 (AOAC, 2005); the obtained number indicated crude protein content:

Percentage of nitrogen=0.1normal acid content/Sample weight×100.

Crude protein percentage=nitrogen percentage×6.25.

### 2.13 Determination of ash content

A muffle furnace (Isuzu) was used to measure percentage of ash. First, 10 g of sample, which had been previously dried and weighed in the oven, was placed in a Crucible and then, placed in an electric oven at 500°C for 12 h. Then, they were placed in a desiccator for 30 min to cool. The Crucible was placed in the desiccator and weighed (AOAC, 2005). The weight of remain material was determined based on the following formula:

$\text{Percentage of}\;\mathrm{ash}=\left(\mathrm{Ash}\;\text{weight}/\text{Initial sample weight}\right)\times 100.$

### 2.14 Determination of carbohydrate content

Carbohydrate content was determined based on difference calculation: [%carbohydrate = 100−(%moisture + %ash + %crude protein + %fat)].

### 2.15 Energetic value

The energetic value was determined indirectly using Rubner's coefficients for aquatic organisms: 9.5 kcal/g for fat, 5.65 kcal/g for proteins (Winberg, 1971), and expressed in kcal/g and kJ/g wet mass as described by Eder and Lewis (2005).

### 2.16 Physicochemical parameters

Physicochemical parameters were recorded daily at the end of experiment. The average of the data was calculated.

### 2.17 Statistical analysis

SPSS software was used to statistically analyze the data, and the normality of data distribution was checked by Kolmogorov–Smirnov method. Using one‐way ANOVA test, the presence or absence of differences between treatments was examined and after observing a significant difference, Duncan test was at 95% confidence level to check the significance of differences between treatments.

## 3 RESULTS AND DISCUSSION

The present study demonstrated that the inclusion of grape pomace in fish diet changed the growth performance, nutritional indices, and body composition as shown in Tables 1–3. All growth parameters, survival rate, HSI, and VSI showed the highest rate in treatment 3, moisture, crude protein, and carbohydrate contents in treatment 2, crude lipid, energy and CF in treatment 1, FCR, and pH in control.

**TABLE 1:** Comparison of average growth and survival indices in common carp fed with different levels of grape pomace.

Treatments	Control (0)	G1 (5%)	G2 (10%)	G3 (15%)
WG	17.46 ± 0.97^b^	15.59 ± 0.46^b^	16.63 ± 0.052^b^	31.36 ± 0.86^a^
DWG	29.11 ± 1.61^b^	25.98 ± 0.76^b^	27.72 ± 0.87^b^	52.27 ± 1.26^a^
BWG	15.62 ± 0.68^b^	14.04 ± 0.46^b^	14.85 ± 0.58^b^	28.25 ± 0.64^a^
SGR	0.10 ± 0.003^b^	0.10 ± 9.81^b^	0.10 ± 0.003^b^	0.18 ± 0.006^a^
FCR	1.41 ± 0.003^a^	1.35 ± 0.024^b^	1.38 ± 0.013^ab^	1.34 ± 0.003^b^
SR	95.55 ± 0.57^ab^	88.88 ± 2.22^ab^	91.10 ± 2.22^b^	97.77 ± 2.22^a^

*Note*: Different letters in each row indicated a significant difference between the treatments (mean ± SD; *p* < .05).

### 3.1 Growth performance and survival indices

Based on the data statistical analysis, treatment 3 (concentration 15%) had the highest mean body weight gain compared to the other treatments (*p* < .05), but the difference was significant between control, G1 (5%) and G2 (10%) treatments (*p* < .05). Table 1 shows that average body weight gain (AWG) in common carp was affected by different levels of grape pomace on control treatment, G1 treatment (5%), G2 treatment (10%), and G3 treatment (15%), with a significant difference (*p* < .05). Table 1 shows that daily weight gain in the carp fish increased in G3 treatment (15%). The treatment G3, with 15% grape pomace, showed a significant difference between control and the treatments 5% and 10% (*p* < .05; Table 1).

The diet containing 15% grape pomace was performed almost twice as well as the other treatments and resulted in higher weight gain, daily weight gain, and specific growth rate. The improved improvement in the 15% grape pomace diet was due to other factors such as feed rate. The results obtained in Table 1 for the percentage of body weight gain showed that G3 with concentration of 15% had highest significant difference compared to the control and G1 (5%) and G2 (10%) treatments (*p* < .05). According to the results, the percentage of specific growth rate in G3 with concentration of 15% was highest compared to the control and G1 (5%) and G2 (10%), respectively (*p* < .05; Table 1). There was no significant difference between treatments for survival rate (*p* < .05), and the survival rate for G3 was the highest, while survival percentage in G1 observed was the lowest (*p* < .05). The results showed that the CF in the present study had a significant difference between different treatments (*p* < .05). The highest rate was for G3 and the lowest was observed in G1 (Table 1).

According to the results in Table 2, lowest FCR was obtained in G3 and the highest was related to the control with a significant difference (*p* < .05). The hepatosomatic index did not show significant difference between the experiment treatments, but the control treatment showed lower liver index compared to the other treatments while the treatment 3 showed highest liver index (Table 2).

**TABLE 2:** Comparison of average morphological parameters in common carp fed with different levels of grape pomace.

Treatments	Control (0)	G1 (5%)	G2 (10%)	G3 (15%)
HIS	3.90 ± 0.057^b^	3.93 ± 0.024^b^	4.03 ± 0.033^ab^	4.10 ± 0.057^a^
VSI	8.87 ± 0.396^b^	8.74 ± 0.493^b^	9.48 ± 0.423^ab^	10.78 ± 0.351^a^
CF	1.60 ± 0.57^ab^	1.39 ± 0.10^a^	1.52 ± 0.03^ab^	1.89 ± 0.03^a^

*Note*: Different letters in each row indicated a significant difference between the treatments (mean ± SD; *p* < .05).

There was a significant difference in VSI between experiment treatments fed with different concentrations of grape pomace, in which treatment 3 had the highest index (*p* < .05) (Table 2).

### 3.2 Body composition

The results showed that there was a significant difference in moisture content between experiment treatments fed with different levels of grape pomace, in which control treatment had lowest and treatment 3 had highest (*p* < .05).

There was a significant difference in pH level between experiment treatments, in which control treatment had the highest pH and G2 had lowest pH (*p* < .05). For ash and crude lipid contents, there was a significant difference, which for ash, G1 treatment had lowest, and G3 treatment had highest, but for crude lipid, G1 treatment had highest and G3 treatment had lowest (*p* < .05). For crude protein content, there was a significant difference between experiment treatments, in which G2 treatment had highest and G1 treatment had lowest (*p* < .05). The results showed that there was a significant difference in carbohydrates content, in which control treatment had highest and G2 treatment had lowest (*p* < .05). For energetic values, there was a significant difference between treatments, in which G1 treatment had highest and G3 treatment had lowest (*p* < .05; Table 3).

**TABLE 3:** Body composition (%) of carp fish fed diets with different grape pomace levels.

Grape level (g/kg food)	G1 (50)	G2 (100)	G3 (150)	Control (0)
Moisture (%)	74.17 ± 0.47^b^	75.73 ± 0.23^a^	76.13 ± 0.76 ^a^	73.3 ± 1.47^c^
Crude protein (%)	18.03 ± 0.23^c^	18.17 ± 0.21^a^	18.1 ± 0.17^b^	18.1 ± 0.17^b^
Crude lipid (%)	6.03 ± 0.44^a^	4.33 ± 0.24^b^	3.9 ± 0.81^b^	5.53 ± 74^a^
Ash (%)	1.08 ± 0.12^d^	1.16 ± 0.27^b^	1.22 ± 0.16^a^	1.12 ± 0.06^c^
Carbohydrate (%)	0.69 ± 0.15^a^	0.61 ± 0.11^a^	0.64 ± 0.02^a^	1.28 ± 0.48^b^
pH	6.37 ± 0.05^b^	6.29 ± 0.09^c^	6.39 ± 0.03^b^	6.45 ± 0.05^a^
Energy (kcal)	132.8 ± 4.72^a^	117.2 ± 2.85^b^	113/1 ± 7.11^b^	130.8 ± 7.43^a^

*Note*: Different letters in each row indicated a significant difference between the treatments (mean ± SD; *p* < .05).

The low water quality conditions reduced feed intake, increased FCR values, and reduced survival rates (Björnsson & Ólafsdóttir, 2006; Santos et al., 2010). Physicochemical parameters such as temperature, DO, and pH remained within the acceptable range for growing fish as shown in (Table 4).

**TABLE 4:** Physicochemical parameters of water in the carp growing environment in the present study.

Parameters	pH	EC (μmho/cm)	DO (mg/L)	Hardness (mg/L)	Temperature (°C)
Level	7–8	2300	6.5–7	140–170	23–27

The fish aquaculture industry and fish nutrition is considered one of the biggest challenges in aquaculture that has an important role in production costs (Ahmad et al., 2018; Silva et al., 2007). Therefore, feeding rate affects fish growing, to reduce the feeding loss and preservation of water quality, hence reducing the water exchange and regulation of the consumed feed. The effects of the feeding rates vary on water quality, growth parameters, feed efficiency, survival rate, and also the composition of fish muscle.

The results of the present study show that the best growth performance was recorded in fish fed with 15.0% BWG with feedings of three times daily after a 56‐day trial. A 15.0% BWG feeding rate showed significantly higher SGR, WG, and the best VSI and HSI. But increasing the feeding level till 5% BWG resulted in reducing the growth performances.

In regard to feed utilization parameters, the best FCR was achieved with fish fed at 10% daily, while it can be said that the feed utilization parameters and the growth indicate agreed to T3 which has the optimum feeding rate. Absolutely, an increasing feeding rate at level higher than the optimum level results in decrease in FCR; it could be observed that G3 had the best option followed by G2 and G1.

This work showed that grape pomace was a suitable food supplement in the fish diet. It was determined that grape pomace had a positive effect on growth performance; the administered dose of 150 g/kg feed (G3) in particular positively affected all growth parameters similar to HIS and VSI indices. Similar to our study, a study on tilapia and carp (Maqsood et al., 2009) which for the fish tilapia‐fed diet supplemented with ginseng had a positive growth performance (Ashraf & Goda, 2008), and on carp (Nya & Austin, 2009) and on rainbow trout with garlic (*Allium sativum*) consumption. Morphological indicators such as CF, HSI, and VSI are used to determine the natural and physiological status, which can provide evidence about development, health, energy reserves, and the potential of fish to survive ecological stress. In uncomfortable and critical environmental conditions, fish liver usually loses more energy and is smaller in size. Morphological statistical analysis in the present study showed that increased significantly with increasing feeding rate. This observation was in agreement with the report of Mohammed et al. (2017) who found that HSI did not differ among the treatments by the change of feeding level; in the same trend, Du et al. (2006) ascertain that HSI did not affect with increasing feeding rate.

Experiments (Pena et al., 2019) showed a positive effect on digestibility in rainbow trout fry that fed with grape pomace at level of up to 180 g/kg with no negative effects on growth and feed with grape pomace at level of up to 60 g/kg. Their findings highlighted the importance of basic research to determine the potential use of grape pomace in aquaculture. In this sense, the inclusion of grape pomace in lamb feed improved feed efficiency and muscle tenderness, probably from the attenuation of oxidative stress (Poli et al., 2003; Zhao et al., 2018).

In the present study, the use of different levels of grape pomace in the fish diet showed that the mean body weight gain in G3 (15%) was significantly different compared to the control which increased the fish weight. Also, for daily weight gain, body weight gain percentage and specific growth rate percentage found an increase in G3 (15%), which was agreement with the results of Abdulrahman (2016), which was related to the effect of different levels of grape seed in the finger carp and the typical carp (Zamani Moafi, 2017); replacing the fish oil with grape pomace oil in the salmon diet showed significant increase in the final weight, body weight, and specific growth rate of fish up to 50%, which was in agreement with the results of the present study. In a study, the effect of grape pomace at different levels on ordinary carp finger fish was investigated and observed that the fish's final weight at all levels compared to the control had a significant difference (Abdulrahman, 2016), which was different from the results of the present study.

Another study was carried out on the effects of black grape pomace with 2.5%, 5%, 7.5%, and 10% in the diet on the growth performance of common carp, and the results showed that the addition of grape pomace in higher amounts had a positive effect in growth indices with significant different compared to the control (Nader & Abdulrahman, 2019). These results were in agreement with the results of the present study. Nader and Abdulrahman (2019) proved that the inclusion of black grape pomace in fish diet has affected the growth performance as it increased the total weight gain, relative growth rate, and specific growth rate. In this study, the inclusion of grape pomace concentrate has changed the weight gain compared with fish fed with the control diet. However, feed efficiency was improved by the addition of grape pomace concentrate in comparison with fish fed with control diet. The present study results were in agreement with the results of Zhai et al. (2014), which shows that the final body weight and weight gain of tilapia (*Oreochromis niloticus*) were significantly higher than the control. G3 (15%) had the highest survival rate, but there was no significant difference between the experiment treatments. The improved performance in the 15% grape pomace inclusion diet may be due to some other factors, such as feed rate. Based on FCR, we guess that 15% grape pomace treatment was provided almost twice as much feed as the other treatments.

In a study, the average survival rate of shrimps in all experiment groups was not significantly different from each other (Niyamosatha et al., 2015), which was different from the present study results. In the present study, changes in nutritional index such as FCR were significantly different, but there was no significant difference between treatments for liver index.

The growth and weight gain rate are directly related to animal capability for digestion and absorption of nutrients (Magouz et al., 2020). Medicinal plants have been used to improve nutrition in human for a long time, and their use has drawn considerable attention in aquatic animals (El‐Deep et al., 2019; Saleh et al., 2019; Shekarabi et al., 2020).

The inclusion of the extract of grape pomace improved the growth performance of common carp. The optimum diet GSE was 20–30 g/kg diet for improving the performances of common carp. Therefore, GSE could be used as a promising natural antibacterial additive for protecting the fish from infection (Mehrinakhi et al., 2021).

Grape pomace has limited use as a feed ingredient due to consequent reduction in energy availability and its low digestibility (Baumgarte et al., 2007; Feedipedia‐Animal Feed Resources Information System, 2016; Spanghero et al., 2009). It was shown that lower digestibility may be due to the high fiber and tannin content in grape pomace, which can increase the formation of tannin and indigestible protein complexes, prevent the growth, and inhibit proteolytic enzyme activity (Zhao, 2007). Therefore, fat content in G3 and G4 treatments, protein content in G1 treatment, and energy value in G3 and G2 treatments decreased compared to the control. One researcher reported that the addition of grape seed to fish diet significantly affected protein, fat, ash, and moisture percentage in fish meat (Abdulrahman, 2016). The role of polyphenolic compounds in diet may be some adverse metabolic effects due to lower efficiency of nutrients particularly protein, inhibition of digestive enzymes, and increased excretion of endogenous protein; polyphenols bind to protein (Abdulrahman, 2016; Brenes et al., 2010), and this was opposite with our results.

Increase in the production efficiency with good quality is one of the most important goals in aquaculture. Food additives are one of the strategies, because they can promote growth and development of fish and can also play an important role in improving fish health and increasing the muscle quality by increasing resistance to pathogens and stress, in addition to provide the necessary nutrients (Aberoumand et al., 2019).

In the present research, the composition of fillets (Table 3) was affected by the difference in feeding level. It was observed that the moisture content changed with the change in feeding level. Regarding the effect of feeding level on whole‐body growth, protein content was largely unaffected by increasing feeding levels. However, fat level decreased significantly with increasing feeding levels. Similar results were recorded by Mohammed et al. (2017); they illustrated that the whole fish body content of protein did not affected with increasing feeding levels. Generally, in the present study, using 15% grape pomace in the fish diet significantly increased growth performance, survival, HSI, and VSI indices.

The feeding of the fish with the experimental diet (Table 5) showed that individual carp grow in wide range of rates even on the same diet. With 15 fish per treatment tank with three replicates, the growth performance data showed could be the result of a few carp with elevated growth rates not directly resulting from the grape pomace. The large difference between the 10% and 15% pomace rates supplies evidence to support that something other than amount of grape pomace is responsible for the difference.

**TABLE 5:** Composition and proximate analysis of the experimental diet.

Ingredient	Percent
Fish meal	58.88
Gelatin	10.00
Cod liver oil	4.82
Vitamin mix	0.80
Mineral mix	3.20
Dextrin	15.30
Cellulose	7.00
Moisture (dry weight)	12.50
Crude protein (wet weight)	34.90
Crude lipid (wet weight)	6.00
Ash (wet weight)	4.1
Energy (kcal/g)	260.73

One of important resources of procyanidins and phenols, catechins, and epicatechins is Grape (*V. vinifera*) (Arslan et al., 2018; Lange et al., 2014; Mousavi et al., 2020), which present antimicrobial, anti‐mutagenic, anti‐inflammatory, and antioxidant properties (Nowshehri et al., 2015; Peixoto et al., 2016; Yu & Ahmedna, 2013). Previous studies have shown the beneficial effects of diets with extracts of grape seed for fish. The supplementation of extract of grape seed contributed to growth performance (Arslan et al., 2018; Lange et al., 2014; Mousavi et al., 2020, 2021), immune responses (Mehrinakhi et al., 2021; Mousavi et al., 2021), antioxidant status (Arslan et al., 2018; Mousavi et al., 2020), and disease resistance (Lange et al., 2014; Mehrinakhi et al., 2021) in fish. Crude grape extracts are a by‐product of the processing of juice, formed by the pulp. It can be a viable alternative in practical fish diets for omnivorous fish, in addition to contributing to the sustainable development of aquaculture, due to the use of waste derived from the production of grape juice, which could be discarded in the environment, contaminating soils, and water courses.

## 4 CONCLUSIONS

It can be concluded that using 15% concentration of grape pomace in the fish *C. carpio* diet had a positive effect on nutrition and growth indices. Present study showed that using concentration of 15% grape pomace in the fish diet increased the weight of fish, while the 15% treatment had a higher survival rate than the control treatment. It is suggested that using the grape pomace obtained from juice industry for feeding the carp was useful. In general, different ratios of grape pomace had different inductions on growth and body composition parameters. Based on data obtained and due to the positive effect of grape pomace on growth and survival indices and its cost‐effectiveness, a diet containing 15% of grape pomace (150 g/kg food) is recommended for common carp.

## AUTHOR CONTRIBUTIONS

**Barzan Mahmoodi:** Formal analysis (equal); investigation (equal); methodology (equal); resources (equal); software (equal). **Ali Aberoumand:** Conceptualization (equal); data curation (equal); formal analysis (equal); funding acquisition (equal); investigation (equal); methodology (equal); project administration (equal); resources (equal); software (equal); supervision (equal); validation (equal); visualization (equal); writing – original draft (equal); writing – review and editing (equal). **saeed Ziaei‐nejad:** Supervision (equal). **Sadra Seyyedi:** Investigation (equal); methodology (equal).

## FUNDING INFORMATION

This study was funded by the Behbahan Khatam Alanbia University of Technology, Behbahan, Iran.

## CONFLICT OF INTEREST STATEMENT

The authors have declared no conflict of interests for this article.

## Data Availability

The datasets used are available from the corresponding authors upon reasonable request.
